# Multichannel terahertz quasi-perfect vortex beams generation enabled by multifunctional metasurfaces

**DOI:** 10.1515/nanoph-2022-0270

**Published:** 2022-07-14

**Authors:** Wanying Liu, Quanlong Yang, Quan Xu, Xiaohan Jiang, Tong Wu, Jianqiang Gu, Jiaguang Han, Weili Zhang

**Affiliations:** Center for Terahertz Waves and College of Precision Instruments and Optoelectronics Engineering, and Key Laboratory of Optoelectronic Information Technology (Ministry of Education), Tianjin University, Tianjin 300072, China; School of Physics and Electronics, Central South University, Changsha 410083, China; School of Electrical and Computer Engineering, Oklahoma State University, Stillwater, Oklahoma 74078, USA

**Keywords:** dielectric metasurfaces, multifunction devices, quasi perfect vortex beam, terahertz

## Abstract

Vortex beams carrying orbital angular momentum (OAM) open a new perspective in various terahertz research. Multichannel and divergence controllable terahertz vortex beam generation holds the key to promoting the development of OAM related terahertz research. Here, we introduced and experimentally demonstrated quasi-perfect vortex beam (Q-PVB) with a controllable divergence angle independent of the topological charge and multichannel Q-PVBs generation with all-dielectric multifunctional metasurfaces. By superimposing specific phase functions together into the metasurfaces, multiple vortex beams and four-channel Q-PVBs with different topological charges are generated as well as focused at separated positions. High resolution characterization of terahertz electric field shows the good quality and broadband properties of Q-PVBs. Interestingly, compared with conventional perfect vortex beam; Q-PVB displays a smaller divergence angle and thinner ring width. The metasurfaces proposed here provide a promising avenue to realize multichannel vortex beams generation in compact terahertz systems; benefiting OAM related researches such as mode division multiplexing, vortex beam related plasmonic enhancement and spinning objective detection.

## 1 Introduction

Vortex beam that carries orbital angular momentum (OAM) and exhibits spiral phase front of exp(i*lφ*) (*φ* is the azimuthal coordinate in the transverse plane and *l* is the topological charge) has been exploited as the new dimension for light manipulation in addition to amplitude, wavelength, polarization, and momentum [1]. In the visible domain, vortex beams have played important roles in mode division multiplexing (MDM), quantum information processing, and spinning object detection [2–4]. While for terahertz wave, vortex beams are vital for future research of magnetism, plasmonics, and quantum physics [5–7]. More importantly, vortex-beam based MDM could greatly increase the transmission capacity of terahertz communications, enabling terahertz wireless connection to meet the exponentially increasing data transmission demand in the future [8]. In the applications mentioned above, the generation of multiple vortex beams undoubtedly is the corner stone [9–12]. While comparing with the visible domain, related study in the terahertz band is far from enough and encounters two major difficulties. Firstly, the generation of multiple terahertz vortex beams by many spiral phase plates will not only make the system bulky and complex but also decrease the energy efficiency due to the insertion loss [13, 14]. Secondly, the divergence angle of the vortex beam dependent on the topological charge is also a challenge for designing a miniaturized and integrated multichannel OAM receiver in an MDM system. When using vortex beams in the surface plasmon excitation and spinning objective detection [15, 16], it is desirable to generate vortex beams with dark centers unaffected by topological charges as well. Although perfect vortex beam (PVB) proposed in the visible domain can help to solve this problem [17–19], this method alone is impossible to suitable in various scenarios and the additional lenses and axicons required for PVBs generation are unfavorable for the integration of terahertz system [20].

Hence, an alternative way of generating multiple vortex beams simultaneously is to explore the wavefront manipulation capability of multifunctional metasurfaces [21–23] based on the Pancharatnam–Berry phase (PB phase) [24, 25]. In which the orientation of meta-atom delivers the circular polarization selected phase change linearly proportional to the rotation angle. More importantly, a multifunctional metasurface can replace several bulky components with a thin artificial layer that is ideal for miniaturized and integrated systems [26–29]. The integration of multichannel metasurface with dynamic system [30, 31] may further improve the flexibility of terahertz devices. Multichannel vortex beam generators based on the PB-phase metasurface have been reported in the visible domain [32, 33]. As for the terahertz band, on account of distinctions in raw material response and experimental characterization, only sporadic recent works have shown encouraging advances in this promising direction [34, 35]. More efforts are still required on metasurface-based terahertz vortex beam generators designed especially for OAM related functions such as mode division multiplexing [36], holography [37], and surface plasmon excitation [38]. Moreover, reported metasurfaces for terahertz vortex beams generation [39, 40] have not yet dealt with the strong dependence of the beam divergence angle on the topological charge.

Here, we propose so called quasi-perfect vortex beams (Q-PVBs) that are a new concept to realize vortex beams with a controllable divergence angle independent of topological charges. In addition, multichannel terahertz Q-PVBs generation is also theoretically and experimentally demonstrated based on all-dielectric multifunctional metasurfaces for comparison. We employed the PB phase to build wavelength-insensitive synthetic-phase metasurfaces via incorporating the functions of beam deflection, focusing and Q-PVBs generation. Both two- and four-channel terahertz vortex beams generation with different topological charges are validated by the Rayleigh–Sommerfeld diffraction integral and the experimental measurement using our home-built terahertz near-field time-domain spectroscopy system. The same design concept could be extended to more channels and larger topological charges. More interestingly, the proposed Q-PVB has a smaller divergence angle and thinner ring pattern than PVB with the same topological charge. The proposed Q-PVB based on multifunctional metasurfaces provides a novel avenue for OAM applications with multiple topological charges and stable spot diameters. And the multichannel terahertz Q-PVB generation demonstrated here will pave the way for developing a robust, convenient, and broadband platform for OAM-related applications in the terahertz band.

## 2 Materials and methods

The divergence angle of the vortex beam is mainly determined by its topological charge, which limits the applications of the OAM modes in the terahertz band, especially for OAM multiplexing. Here, we introduce the concept of Q-PVB, which resembles the PVB that has stable annular intensity distributions for different topological charges, while Q-PVB has smaller divergence angle and thinner ring pattern than PVB with the same topological charge. The Q-PVB is the Fourier transform of the reversed BG beam produced by the negative axicon, and the phase profile of a spatial phase plate for the generation of Q-PVB can be defined by:

(1) $$\begin{array}{ll} \phi_{\text{quasi}-\text{perfect}} & =\phi_{\text{vortex}}\left(x,y\right)+\phi_{\text{negative axicon}}\left(x,y\right)\\ & \;+\phi_{\text{lens}}\left(x,y\right), \end{array}$$

here *x* and *y* are the space coordinates, $\phi_{vortex}=l\arctan\left(y/x\right)$ is the phase distribution of a spiral phase plate for vortex beam generation. $\phi_{lens}=2\pi \left(\sqrt{f^{2}+x^{2}+y^{2}}-f\right)/\lambda$ is the phase distribution of the focal lens. *f* is the focal length and *λ* denotes the wavelength. $\phi_{\text{negative axicon}}=-2\pi \sqrt{x^{2}+y^{2}}/D$ is the phase distribution of a negative axicon [41], where *D* represents its period.

**Figure 1:**
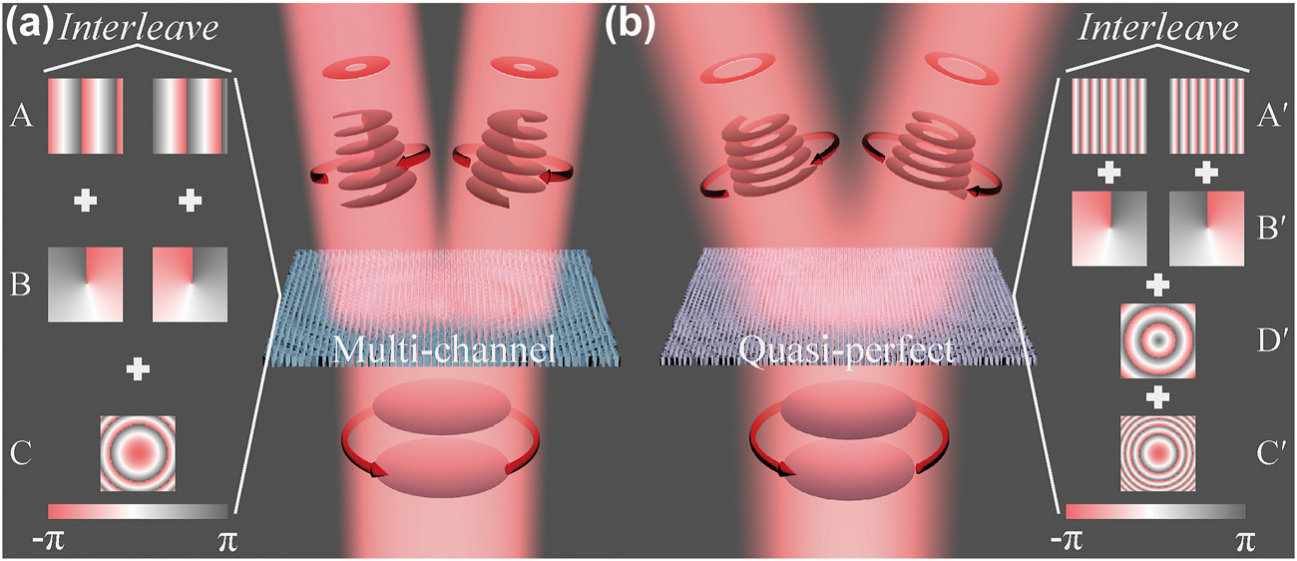
Multi-channel (a) and quasi-perfect (b) vortex beams generation based on all-dielectric synthetic-phase metasurfaces. The phase distribution of metasurfaces A(A′), B(B′) and C(C′) correspond to beam deflection, OAM mode conversion and beam focusing (from up to down). The metasurface D′ in the inset of this figure is designed to replace the negative axicon. The red arrows represent left circular polarization (anticlockwise) and right circular polarization (clockwise). The circular and spiral plane denotes the plane and spiral phase fronts of terahertz waves.

To realize multichannel Q-PVBs generation based on multifunctional metasurfaces and demonstrate the advantage of Q-PVB over ordinary vortex beams, we firstly studied the generation of two-channel vortex beams and Q-PVBs with topological charges of *l* = 1 and −1. All the metasurfaces were designed by superposing several phase profiles with specific functions into one shared-aperture meta-atom array by interleaving arrangement [42]. Here, we use phase discretization to transfer the continuous phase profile to the phase grids and the interval of two adjacent grids is the period of meta-atoms. Figure 1(a) illustrates the generation of two-channel ordinary vortex beams with topological charges of *l* = 1 and −1 (use Metasurface I). As shown on the left, the distribution of the metasurface is the superposition of phase profiles *ϕ*_A_, *ϕ*_B_, and *ϕ*_C_, and the *ϕ*_*ij*_ can be defined by:

(2) $$\phi_{ij}\left(x,y\right)=\left\{\begin{array}{cc} -2\pi \frac{x}{P}+l_{1}\arctan\left(\frac{y}{x}\right)+\frac{2\pi }{\lambda }\left(\sqrt{f^{2}+x^{2}+y^{2}}-f\right) & i=j\\ 2\pi \frac{x}{P}+l_{2}\arctan\left(\frac{y}{x}\right)+\frac{2\pi }{\lambda }\left(\sqrt{f^{2}+x^{2}+y^{2}}-f\right) & i\neq j, \end{array}\right.$$

where *ϕ*_*ij*_(*x,y*) is the accumulated phase in the *i*th row and the *j*th column in a matrix containing *m* × *n* elements (*i* = 1, 2, … , *m*; *j* = 1, 2, … , *n*). Here, *ϕ*_A_= ±2*πx*/*P* represents the phase profiles of beam deflection and *P* is the period of the phase gradient. *ϕ*_B_ and *ϕ*_C_ are used for vortex beam generation and beam focusing, respectively. The key to the interleaving method is the spatial sampling of different phase profiles. Here the “checkboard-like” spatial sampling approach is applied to choose different phase values under the condition of *i* = *j* or *i* ≠ *j*, thus we can deflect two focused vortex beams with different topological charges to opposite directions simultaneously. As for the two-channel Q-PVBs generation, as shown in Figure 1(b), the phase of the metasurface (Metasurface II) is composed of four parts, corresponding to beam deflection (*ϕ*_A^′^_), OAM mode conversion (*ϕ*_B^′^_), beam focusing (*ϕ*_C^′^_), and the phase profile of a negative axicon (*ϕ*_D^′^_). The synthesized phase distribution of Metasurface II then can be expressed as:

(3) $$\phi_{m,n}^{\prime }\left(x,y\right)=\phi_{m,n}\left(x,y\right)-2\pi \sqrt{x^{2}+y^{2}}/D.$$

Taking advantage of the wavelength-insensitive operation and 0-2*π* full phase manipulation capability of the PB phase [43–45], we selected anisotropic all-dielectric square columns to constitute the target phase profile in Figure 1. When the left-circularly polarized (LCP) terahertz wave passes through the meta-atoms, the output beam with opposite helicity (right-circularly polarized, abbreviated as RCP) will be imprinted with a geometric phase *ϕ*_*g*_ = 2*θ* corresponding to the orientation angle *θ* of the anisotropic meta-atom. Hence the target phase distribution of multifunctional metasurface converts to the orientation angle mapping of the meta-atoms. High-efficiency conversion from LCP beam to RCP beam can be obtained by making the meta-atom approximate to a perfect half-wave plate. Here, the selected meta-atom essentially satisfies *t*_*xx*_ = *t*_*yy*_ and *ϕ*_*xx*_ – *ϕ*_*yy*_ = *π*, where *t*_*xx*_ and *t*_*yy*_ are the transmission amplitude under the vertically and horizontally polarized incidence, respectively, *ϕ*_*xx*_ and *ϕ*_*yy*_ represent the corresponding phase delay. We employed CST Microwave Studio to characterize the transmission spectra of the meta-atoms and the multifunctional metasurfaces. All the multifunctional metasurfaces are made of loss-free silicon with a permittivity of *ε* = 11.9. The height of the chosen silicon column is 250 μm and the thickness of the silicon-substrate is 750 μm. The period of the unit-cell is 160 μm and the width and length of the column are 58 and 122 μm, respectively. The simulated transmission spectra of the selected meta-atoms with different rotation angles could be found in Supplementary Information (Figure S1).

## 3 Results

**Figure 2:**
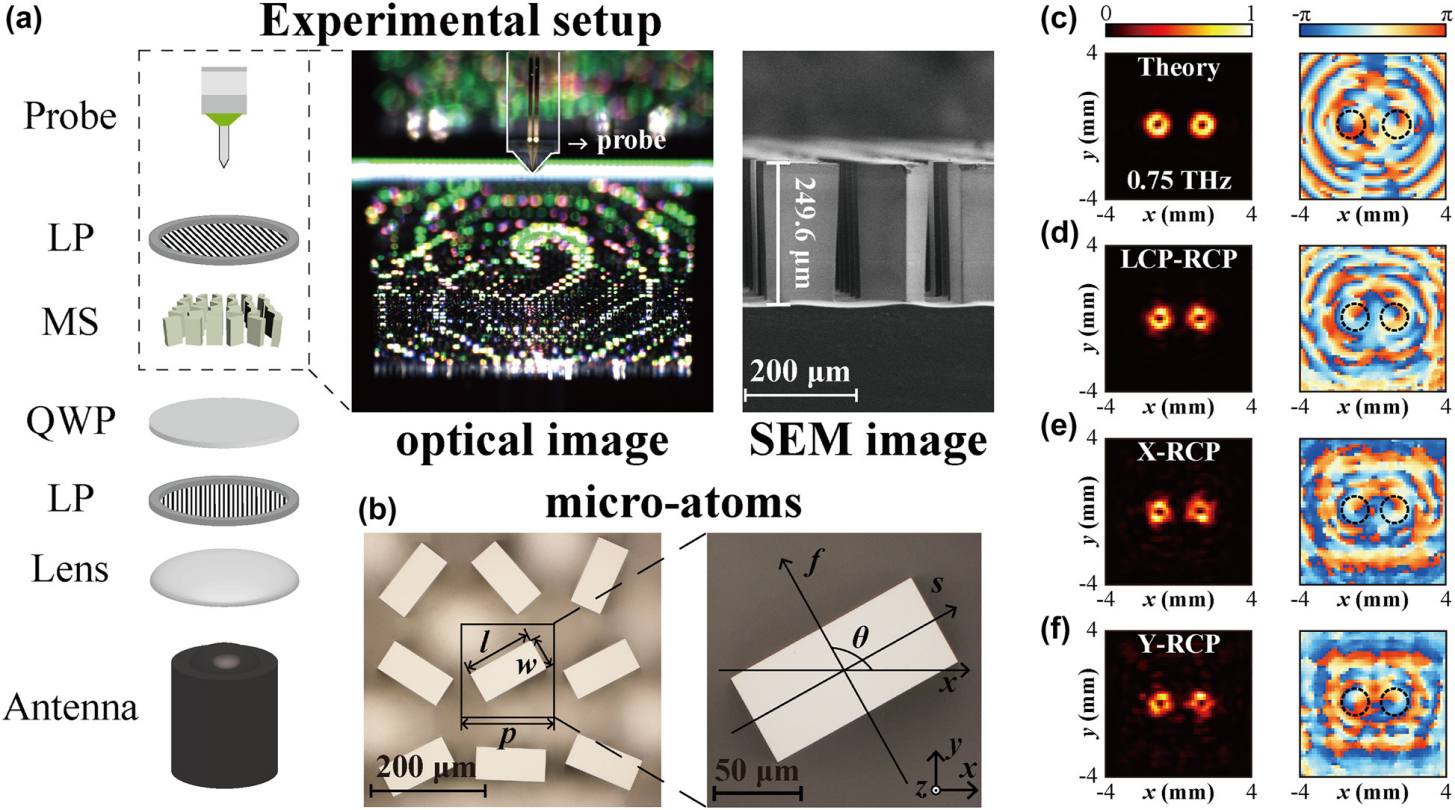
Two-channel vortex beams generation with topological charges of *l* = 1 and −1. (a) Left: schematic illustration of the experimental setup. LP, linear polarizer; MS, metasurface; QWP, quarter-wave plate. Middle inset: the optical image of the fabricated Metasurface I sample and terahertz probe. Right inset: SEM image of the fabricated metasurface. (b) Partially enlarged optical image of the fabricated Metasurface I sample. *p*: period of a unit cell; *l/w*: length/width of the square column; *f*/*s*: fast/slow axis; *θ*: rotation angle. (c) The calculated intensity and phase distribution of proposed Metasurface I with the focal length of *f* = 10 mm. (d) Corresponding measured results with LCP incidence on the measured focal plane (*z* = 9 mm). (e) and (f) Measured results of RCP component under horizontally (*X*) and vertically (*Y*) polarized incidence.

The performance of the PB-phase based metasurface was numerically investigated by the Rayleigh–Sommerfeld (RS) diffraction theory. The RS diffraction formula can be expressed as:

(4) $$U\left(P_{1}\right)=\sum \sum \frac{d}{i\lambda }U_{0}\left(P_{0}\right)\frac{e^{-ikr_{01}}}{{r_{01}}^{2}}dxdy,$$

where *U*(*P*_1_) and *U*_0_(*P*_0_) represent the electric field at point *P*_1_ on the focal plane and point *P*_0_ on the metasurface, respectively; *d* is the distance between the metasurface and the focal plane; *k* = 2*π*/*λ* is the wave vector; *r*_01_ is the distance between point *P*_0_ and *P*_1_. In the calculation, *U*_0_ = *A*_0_ exp(i*ϕ*_*m*_) is defined as the initial incident wave. Here, *A*_0_ = exp[−(*x* ^2^ + *y* ^2^)/*w* ^2^] means the incident beam shows an amplitude distribution of the Gaussian beam and *w* is the waist radius, *ϕ*_*m*_ is the phase distribution of the objective metasurface. Figure 2(c) displays the calculated intensity distribution and the phase distribution of vortex beams generated by Metasurface I at the focal plane of *f* = 10 mm, here the waist radius of the incident Gaussian beam is set to *w* = 4.8 mm, and the period of phase gradient is set to *P* = 3.5 mm for separating the two vortex beams. As shown in Figure 2(c), the focused vortex beams with different topological charges are separated in space as expected, and both the left and right vortex beams have the typical hollow ring-shaped intensity distribution and the spiral phase distribution.

To experimentally generate two-channel vortex beams discussed above, we fabricated the metasurface samples by photolithography and deep reactive ion etching. A 1 mm-thick high-resistance double-side-polished silicon wafer was used to fabricate the metasurface sample. The fabrication can be classified into three steps: first, the square-column patterns were defined by photolithography after the silicon wafer was spin-coated with a 10 μm-thick AZ2070 photoresist. Next, deep reactive ion etching was employed to etch the unwanted silicon areas to a depth of 250 µm, where the remaining photoresist served as a mask to protect the silicon columns. This process consists of two steps: passivation and etching. The former uses C_4_F_8_ to deposit fluorocarbon protective polymer, and the latter etches the silicon with SF_6_, these two steps are alternated until the etching depth meets our design. At last, the metasurface sample was finalized by cleaning the residual photoresist with acetone and acid. The partially enlarged optical image of the completed sample could be seen in Figure 2(b), which displays good etching quality. In the measurement, we used the terahertz near-field time-domain scanning spectroscopy system to demonstrate the feasibility of our multifunctional metasurfaces. As illustrated in Figure 2(a), the terahertz wave emitted by the photoconductive antenna is collimated by a TPX lens. Then a wire-grids based polarizer behind the lens is employed to ensure the high polarizability of the incident terahertz wave. Above the polarizer, we applied a quartz quarter-wave plate designed for 0.75 THz to transform the linear polarized beam into an LCP beam. This circularly polarized terahertz wave impinges the sample from its backside and the transmitted wave is scanned by the terahertz probe mounted on a two-dimension motorized translation stage. Since the near-field probe can only detect linearly polarized electric field, we scanned two orthogonal polarization components along the direction of +45° and −45° to constitute the RCP component. The middle inset of Figure 2(a) displays the detail of the Metasurface I sample laying between the holder and the terahertz probe. The right inset of Figure 2(a) is the cross-sectional image taken with a scanning electron microscope (SEM). The etching depth of the silicon pillar is about 249.6 μm, which matches well with our designed height.

Figure 2(d) shows the measured electric field intensity distribution and the phase distribution of vortex beams at the measured focal plane of *z* = 9 mm. The adopted coordinate system is shown in Figure 2(c), and the upper surface of the metasurface is set as the plane of *z* = 0. The intensity distribution of both two beams displays the complete vortex shapes. Through the measured phase distribution, we can identify that the topological charges of these two vortex beams are *l* = +1 and *l* = −1, which match well with the calculated results from the RS theory. The smaller focal length departure from the calculation mainly comes from the fabrication error and the difference between the real incident beam and the ideal Gaussian incidence adopted in the calculation. In addition to considering LCP incidence, we also investigated the performance of the multifunctional metasurface under horizontally and vertically polarized incident beams. Since the horizontal (*H*) and vertical (*V*) polarization states can be described by a superposition of right-handed (*σ*_+_) and left-handed (*σ*_−_) circular polarization states as $\left|H\right\rangle =1/\sqrt{2}\left(\left|\sigma_{+}\right\rangle +\left|\sigma_{-}\right\rangle \right)$ and $\left|V\right\rangle =1/\sqrt{2i}\left(\left|\sigma_{+}\right\rangle -\left|\sigma_{-}\right\rangle \right)$ , the proposed metasurface can transform the LCP beam into the focused vortex beam while RCP component into the divergent beam. Figure 2(e) and (f) illustrate the intensity and phase distributions of output RCP components under horizontally and vertically polarized incidence, respectively, in which the vortex beams with topological charges of *l* = +1, −1 still could be separated and focused. Hence, in addition to LCP incidence, the metasurfaces can also work under linearly polarized or elliptically polarized incidence but with reduced efficiency.

**Figure 3:**
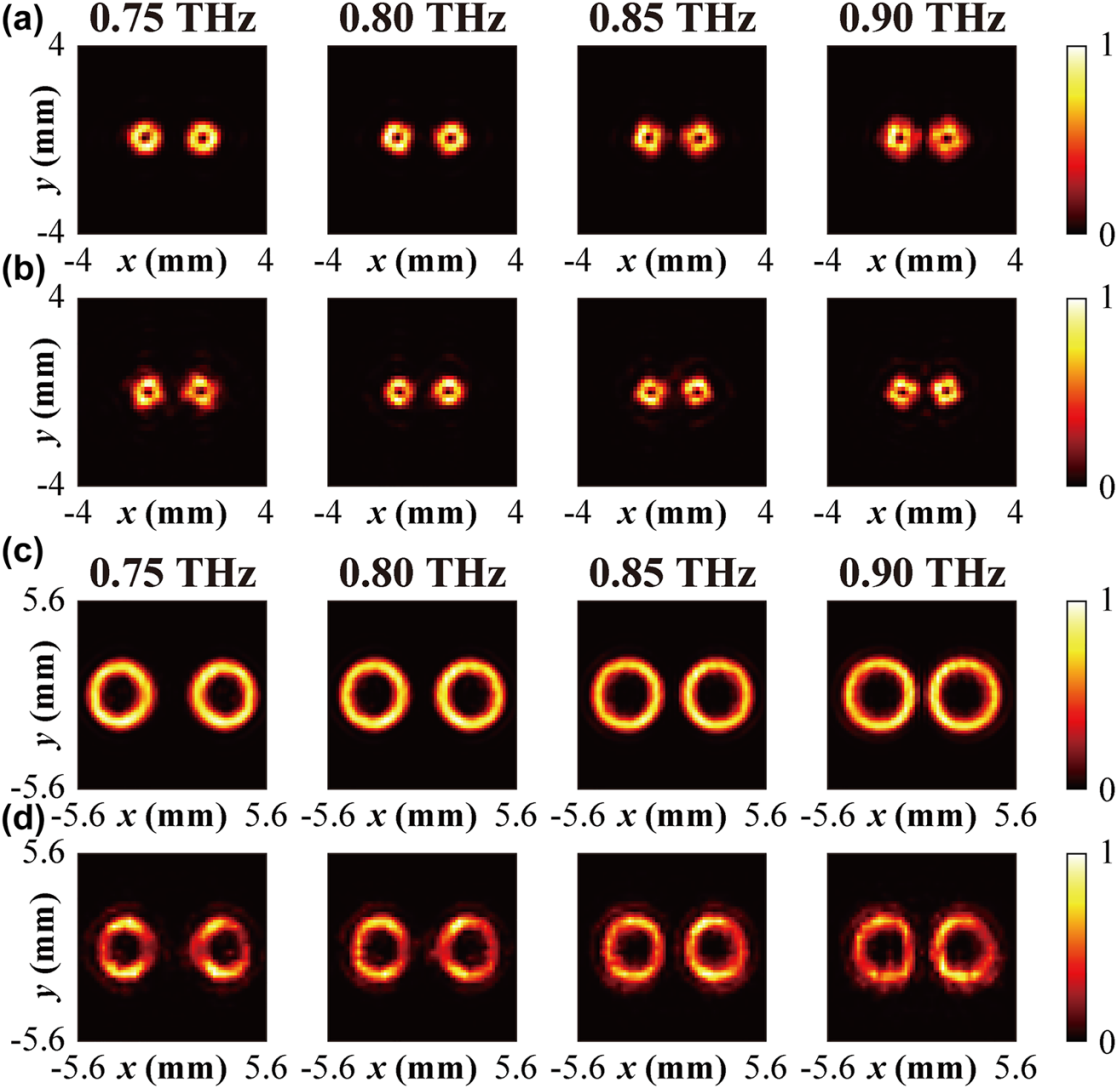
Generation of two-channel vortex beams and Q-PVBs. (a) Calculated and (b) measured intensity distributions for the two-channel vortex beams generation with topological charges of *l* = 1, −1 at 0.75, 0.8, 0.85 and 0.9 THz. (c) Calculated and (d) measured intensity distribution of the focused Q-PVBs with topological charges of *l* = 1, −1 at different frequencies.

Furthermore, considering the wavelength-insensitive feature of the PB phase, we investigated the performance of the multifunctional metasurface out of the target frequency (0.75 THz). Figure 3(a) displays the calculated intensity distribution of vortex beams generated by Metasurface I at the focal plane for 0.75, 0.8, 0.85, and 0.9 THz. All the vortex beams display the typical hollow ring intensity distribution at these frequencies. In the experiment, since the quarter-wave plate was designed for 0.75 THz, we applied another polarizer to replace it and the output RCP wave was obtained by two orthogonal polarizations along with the directions of +45° and −45°. From the measured intensity distribution shown in Figure 3(b), the vortex beams generation and separation could be observed in a broadband frequency range. However, the LCP–RCP conversion efficiency still varies with the operating frequencies since the conversion efficiency dispersion of the meta-atoms. More information could be found in Supplementary Information (see Figure S1).

For the demonstration of two-channel Q-PVBs generation, Metasurface II was designed to generate Q-PVBs with topological charges of *l* = +1 and −1 at 0.75 THz. Here, the period of the negative axicon is selected as *D* = 2.5 mm, the focal length is *f* = 10 mm, and the period of the phase gradient is set to *P* = 1.4 mm. Figure 3(c) shows the calculated electric field intensity distribution of Q-PVBs at the focal plane of *z* = 10 mm. There are two vortex beams that display the perfect annular rings, which prove the validity of our proposed design. These two Q-PVBs at 0.75 THz show a smaller ratio of ring width to beam diameter compared with focused vortex beams generated by Metasurface I. Here the ratio of ring width to beam diameter is 0.26, while for focused vortex beams that is 0.32. In the experiment, the measured intensity profiles of Q-PVBs match well with the calculated results, which could be seen in Figure 3(d). The non-uniform intensity of the measured vortex beams mainly comes from the imperfect field intensity profile of the incident beam. Metasurface II also works at a broadband frequency range, the calculated and measured field intensity distribution of the Q-PVBs at 0.8, 0.85 and 0.9 THz are shown in Figure 3(c) and (d), respectively. All the Q-PVBs with topological charges of *l* = +1 and −1 display the complete annular rings in these frequencies, and the annular ring radius increases with the increment of the frequencies.

**Figure 4:**
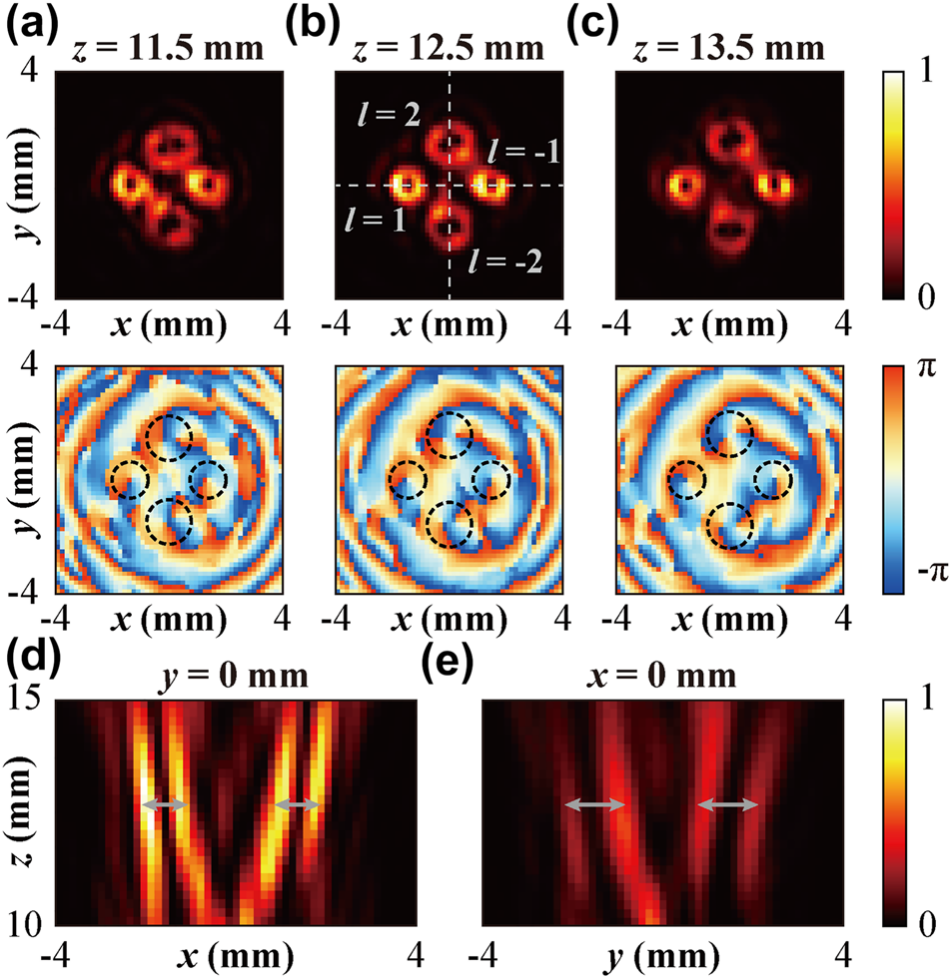
Four-channel focused vortex beams generation with different topological charges (*l* = 1, −1, 2, −2) (a–c) measured electric field intensity and phase distribution of the generated vortex beams at three different *x*–*y* planes. (d) and (e) measured electric field intensities at the *y* = 0 mm and *x* = 0 mm planes.

Furthermore, the generation of multi-channel Q-PVBs with controllable beam divergence and stable annual ring pattern was investigated for topological charges *l* with different absolute values, which is vital for MDM and other multichannel vortex beams applications. First, Metasurface III as a control was designed to generate four-channel ordinary vortex beams with the topological charges of *l* = 1, −1, 2, and −2 at 0.75 THz. The focal length of the vortex beams was set as *f* = 15 mm and the period of the phase gradient is *P* = 3.66 mm. Figure 4(a)–(c) show the measured electric field intensity and phase distributions of the vortex beams at propagation distances of *z* = 11.5, 12.5, and 13.5 mm. From the spiral phase distribution in Figure 4(a)–(c), we found that all the vortex beams with different topological charges were well generated, and the measured LCP–RCP conversion efficiency is 25%. Referring to the donut profiles of the vortex beams, the diameter of the vortex beams with the topological charges of |*l*| = 1 is significantly smaller than that with |*l*| = 2, verifying the beam size of the ordinary vortex beam is dependent on the topological charge. Incidentally, the energy density of the vortex beams with |*l*| = 1 is larger than that for |*l*| = 2. We also scanned the cross-sections of these four vortex beams in the *y* = 0 and *x* = 0 planes (marked by the white dashed line in Figure 4(b)) with the step of 0.16 mm (*x*/*y* direction) and 0.2 mm (*z*-direction), which are shown in Figure 4(d) and (e), respectively. All the vortex beams are focused as well as deflected to a different direction, which corresponds to their propagation behavior is in coincidence with our design.

**Figure 5:**
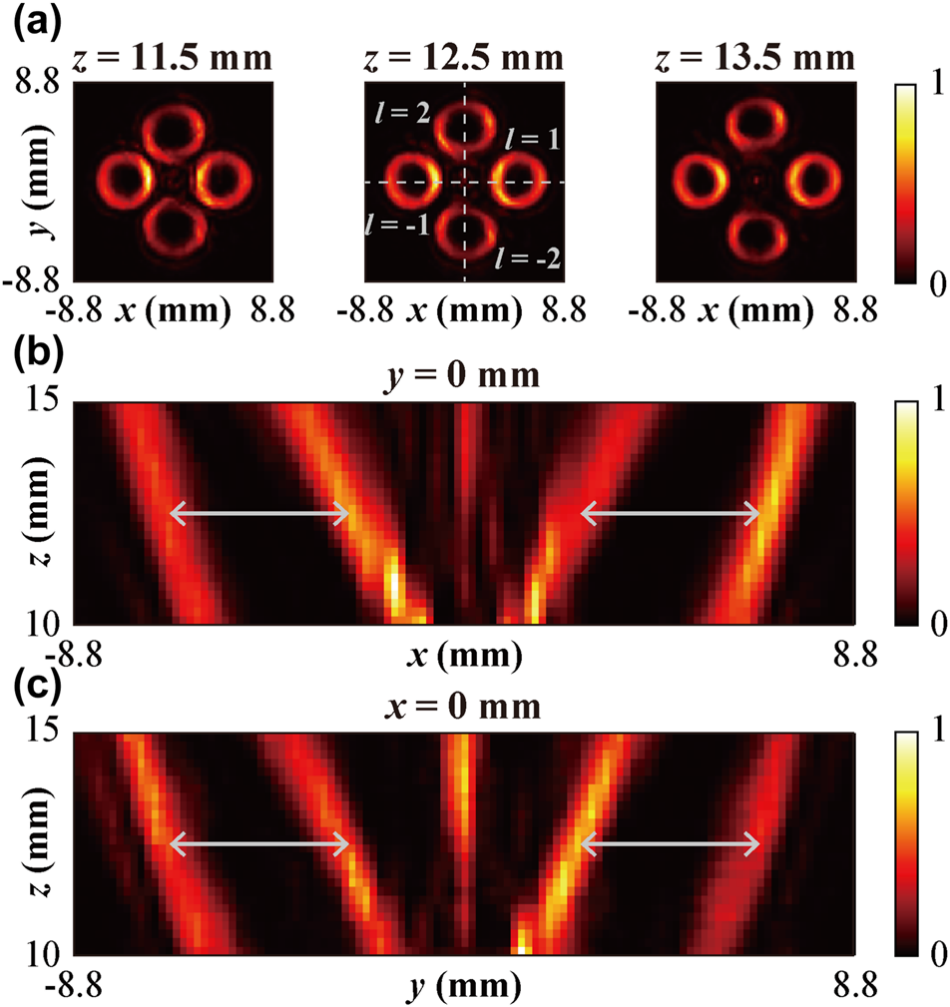
Four-channel Q-PVBs generation with topological charges of *l* = −1, 1, 2, −2 at 0.75 THz. (a) Measured electric field intensity distributions of focused Q-PVBs carrying the topological charges of *l* = −1, 1, 2, −2. The chosen distance of *x*-*y* plane is *z* = 11.5, 12.5, 13.5 mm, respectively. (b) and (c) measured normalized field intensity profiles of *y* = 0 mm plane and *x* = 0 mm plane.

To check the predicted stable spot diameter of Q-PVBs with different topological charges, we designed the Metasurface IV to generate focused Q-PVBs with topological charges of *l* = −1, 1, 2, and −2 at 0.75 THz. Here, the period of the negative axicon is selected as *D* = 2.5 mm, the focal length is *f*= 15 mm, and the period of phase gradient is set to *P* = 1.18 mm. Figure 5(a) illustrates the measured field intensity distributions of the Q-PVBs at the distances of *z* = 11.5, 12.5 and 13.5 mm, and the measured LCP-RCP conversion efficiency is 27%. The interference pattern between an assumed Q-PVB with *l* = 1 and the four-channel Q-PVBs can be used to identify the topological charge of each annular ring by examining the number of spiral branches (More details can be found in Figure S2, Supplementary Information). As shown in Figure 5, the ring diameters of the multi-channel Q-PVBs generated by Metasurfaces IV nearly keep constant independent of the topological charge. Moreover, Figure 5(b) and (c) denote the measured electric field intensity distribution of planes with *y* = 0 and *x* = 0, Q-PVBs maintain the complete ring shape within the measured range of 5 mm. The Q-PVBs proposed here provide a promising way for realizing multi-channel multiplexing to increase the capacity of terahertz multiplexing. For verifying the performance of multifunctional metasurfaces with more channels, six-channel vortex beams and Q-PVBs are also investigated. The calculated and measured results could be found in Figures S3 and S4 (Supplementary Information).

## 4 Discussion

**Figure 6:**
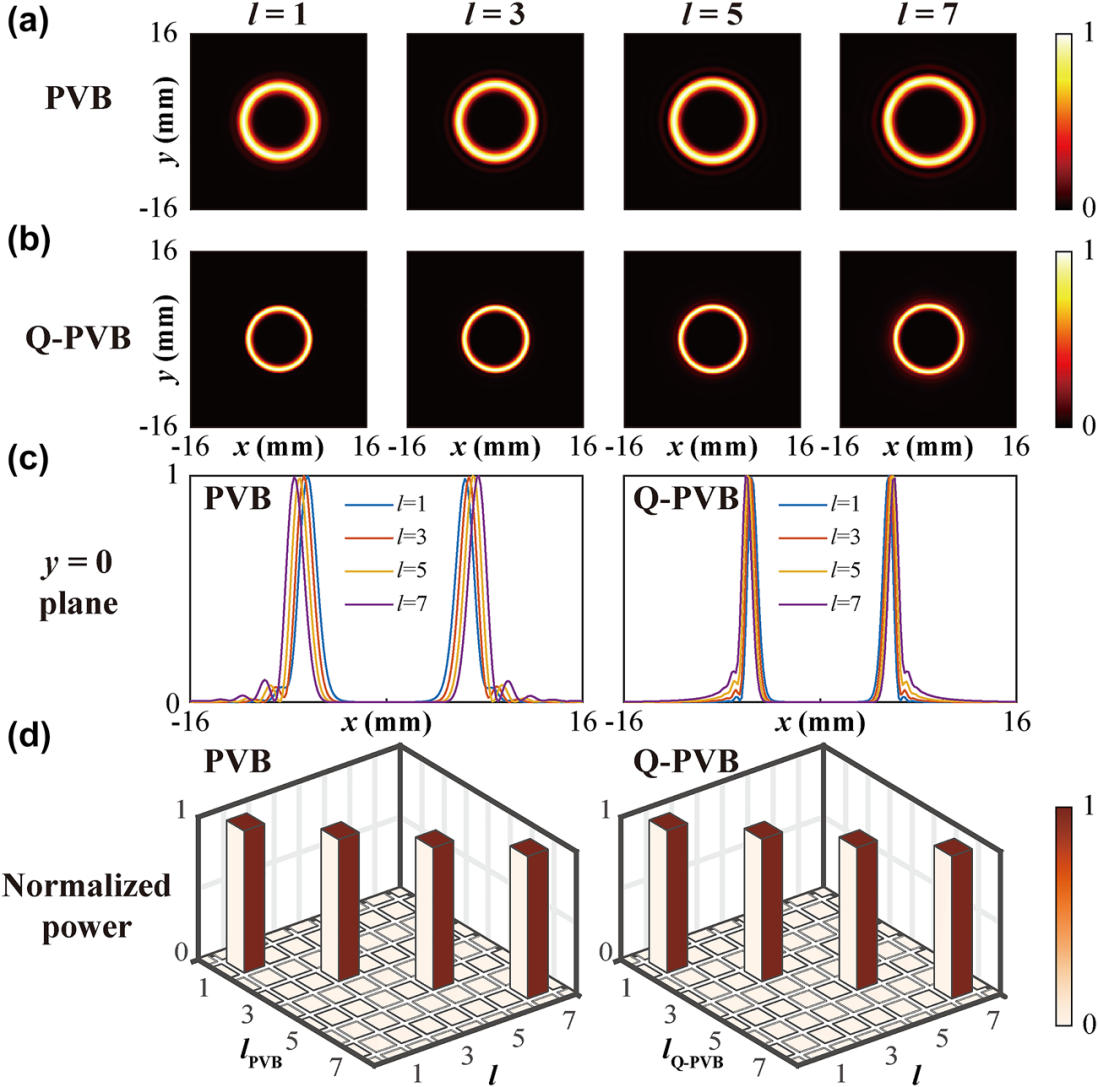
Calculated electric field intensity distribution of PVBs and Q-PVBs with the same *D* and *f* at 0.75 THz. (a) Calculated intensity profiles of PVBs with the topological charges of *l* = 1, 3, 5, and 7 at the focal plane of *z* = 20 mm. (b) Corresponding intensity profiles of Q-PVBs. (c) Cross sections of annular intensity rings of PVBs and Q-PVBs. (d) Mode purities of PVBs and Q-PVBs.

To compare performances between PVB and Q-PVB, here we calculate the intensity distribution of the vortex beams with topological charges of *l* = 1, 3, 5 and 7 at the focal plane using the RS diffraction theory. Here, the period of the negative axicon is chosen as *D* = 1.5 mm, the focal length is *f* = 20 mm and the waist radius of the incident Gaussian beam is *w* = 6 mm. The calculated field profiles at the focal plane of PVBs and Q-PVBs which have the same *D* and *f* are shown in Figure 6(a) and (b). The electric field of the Q-PVB on the focal plane satisfies *E*_Q-PVB_(*r*, *φ*) = exp [−(*r* − *r*_0_)^2^/Δ*r* ^2^]exp (i*lφ*), here (*r*,*φ*) represents the polar coordinate system and Δ*r* is the ring width. Compared with PVBs, Q-PVBs with different topological charges have a stable spot diameter at the focal plane as well, but a smaller spot size, smaller divergence angle and thinner ring width. Furthermore, we also plotted the annular intensity cross-sections of PVBs and Q-PVBs, which are displayed in Figure 6(c). For PVB and Q-PVB with the topological charge of *l* = 1, the spot diameter of PVB is 1.21 times larger than Q-PVB, and the ring width is 1.74 times larger. When the topological charge changes from *l* = 1 to *l* = 7, the spot diameter of PVB increases by 14.98%, while for Q-PVB proposed here it is increases only 7.81%. Besides, the ring width of Q-PVB keeps essentially constant as *l* changes. Our Q-PVBs display a more stable beam size than PVBs, which may lead to a hot topic for MDM applications in terahertz wireless communication. Meanwhile, the use of Q-PVB is not limited to the terahertz regime but also for OAM-related applications in the optical band and microwave band. The thinner ring width of Q-PVB could introduces the larger local-electric field enhancement, which makes it a promising tool in some important application scenarios such as plasmonic enhancement, super-resolution imaging, nanoparticles trapping and remote sensing.

We also applied the Fourier transform analysis to calculate the mode purity of the generated PVBs and Q-PVBs. The phase of the generated vortex *ϕ*(*l*) can be expanded into a sum of fundamental OAM modes as [46, 47]:

(5) $$\phi \left(l\right)=\overset{l=\infty }{\underset{l=-\infty }{\sum }}c_{l}\exp\left(il\varphi \right),$$

here *c*_*l*_ is the coefficient for the Fourier transform:

(6) $$c_{l}=\frac{1}{2\pi }\overset{\pi }{\underset{-\pi }{\int }}\phi \left(l\right)\exp\left(-il\varphi \right)d\varphi .$$

Here we consider the OAM modes range from *l* = 0 to *l* = 8, and the normalized power of each mode can be defined by: $NP\left(l\right)=c_{l}/\sum_{l=0}^{l=8}c_{l}$ , and the calculated mode purities of PVBs and Q-PVBs can be seen in Figure 6(d), the calculated mode purities of both PVBs and Q-PVBs are higher than 99%. Also, we found the ring radius *ρ* of Q-PVB at the focal plane satisfies the relationship *ρ* ∝ *f*/*D* (see Figure S5, Supplementary Information). Furthermore, we also discussed the conversion between Q-PVB and Bessel–Gaussian (BG) beam, Q-PVB converts to BG beam after transmitting a distance of *r*_s_ and the calculated results show *r*_s_ ∝ *f*/*D*. The details can be found in Figure S6, Supplementary Information. The above two relations (*ρ* ∝ *f*/*D* and *r*_s_ ∝ *f*/*D*) could help us to realize arbitrary manipulation of the beam divergence of Q-PVB.

## 5 Conclusions

In summary, we designed and demonstrated a series of all-dielectric synthetic-phase multifunctional metasurfaces based on the PB phase for multichannel vortex beams and Q-PVBs generation. Two- and four-channel vortex beams with different topological charges were demonstrated by the RS diffraction theory and terahertz near-field time-domain spectroscopy system. The metasurfaces display broadband responses from 0.75 THz to 0.9 THz. Moreover, we proposed the concept of Q-PVB with stable spot diameters independent of topological charges and verified these features by the generation of multi-channel with topological charges of *l* = −1, 1, 2, and −2. Notably, Q-PVB displays a smaller beam divergence and thinner ring width than PVB with the same topological charge. Q-PVB is of significance for vortex beams researches with large topological charges and controllable beam divergences, which is not limited to the terahertz regime but also for optical multiplexing techniques. The demonstrated multifunctional metasurfaces provide an efficient platform to improve the integration and compactness of the back-end terahertz device, which will greatly promote the OAM-related applications in next-generation communications, surface plasmon excitation, and terahertz imaging.

## Supplementary Material

Supplementary Material Details
