# Supplementary material for: Multichannel terahertz quasi-perfect vortex beams generation enabled by multifunctional metasurfaces
Source: Nanophotonics. 2022 Jul 14;11(16):3631–40. doi: 10.1515/nanoph-2022-0270 (PMC11501438; doi:10.1515/nanoph-2022-0270)
Supplement: Supplementary file 1 — Supplementary Material Details [file j_nanoph-2022-0270_suppl.docx]

Supplementary Information

Multi-channel terahertz quasi-perfect vortex beams generation enabled by multifunctional metasurfaces

Wanying Liu^1^, Quanlong Yang^2,*^, Quan Xu^1^, Xiaohan Jiang^1^, Tong Wu^1^, Jianqiang Gu^1,*^, Jiaguang Han^1^, and Weili Zhang^3,*^

^1^Center for Terahertz Waves and College of Precision Instrument and Optoelectronics Engineering, Tianjin University, Tianjin 300072, China

^2^School of Physics and Electronics, Central South University, Hunan 410083, China

^3^School of Electrical and Computer Engineering Oklahoma State University

Stillwater, OK 74078, USA

*Corresponding authors. E-mail: [gjq@tju.edu.cn](mailto:*gjq@tju.edu.cn), [quanlong.yang@csu.edu.cn](mailto:quanlong.yang@csu.edu.cn), [weili.zhang@okstate.edu](mailto:weili.zhang@okstate.edu)

Contents

S1. The simulated transmission spectra of the selected meta-atoms 2

[S2. Interference patterns for Q-PVBs generated by metasurface IV 3](#bookmark1)

[S3. Metasurfaces proposed to generate four-channel and six-channel focused vortex beams 4](#bookmark2)

[S4. Metasurfaces proposed to generate four-channel and six-channel Q-PVBs. 5](#bookmark3)

[S5. The relationship between the ring radius of Q-PVB and the two parameters 6](#bookmark4)

[S6. The conversion relationship between Q-PVB and Bessel Gaussian beam 7](#bookmark5)

**S1. The simulated transmission spectra of the selected meta-atoms**

We used CST Microwave Studio to simulate the transmission spectra of micro-atom under x- and y-polarized incidence. The transmission amplitude of the selected square column at 0.75 THz is 0.65 for x-polarization and 0.67 for y-polarization, and the simulated phase difference between two orthogonally linear polarizations is 178.95º, indicating that this micro-column can be approximated as a half wave plate. To verify the relationship between the rotation angle of micro-column and the phase delay, we simulate the transmission spectra of micro-columns with different rotation angles. Here we selected eight rotation angles with the step of 22.5º, the simulated results at 0.75 THz are shown in Figure S1(a), the blue line in the figure corresponding to left-circularly polarized (LCP) wave to right-circularly polarized (RCP) wave converted efficiency and the red line corresponding to the phase delay. Both the rotation angle *θ* and the phase delay *ϕ_g_* are normalized in the figure for comparison. The image inset at the lower right is a micro-column with the rotation angle of 135º. From the simulation results it can be observed that the polarization converted efficiency *η* is around 45% and the full 2π phase control can be realized by rotating the micro-columns inside the unit-cell with the rotation angle of 0-180º, thus the rotation angle of each micro-column in multifunctional metasurface can be obtained from the relation of *θ* = *ϕ_m_* /2, here *ϕ_m_* represents the phase distribution of the objective metasurface. We also simulated the transmission spectra of the selected meta-atoms in a broadband range and analyzed the variation of LCP-RCP conversion efficiency. As shown in Figures S1(b)-S1(d), for the operation frequencies that we choose (0.8, 0.85 and 0.9 THz), the average LCP-RCP conversion efficiency of the selected meta-atoms is 45%, 42% and 34%, respectively.


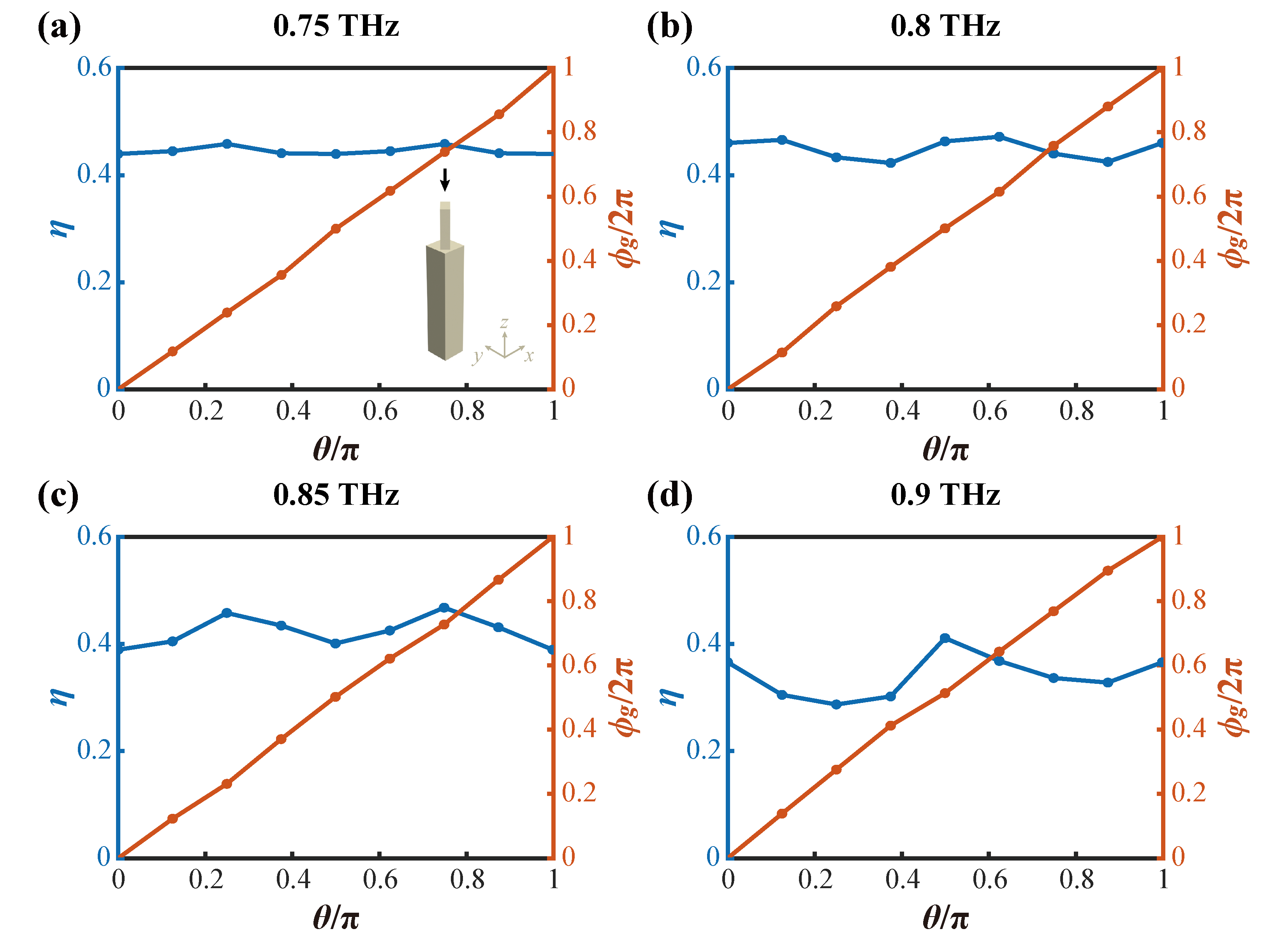


**Figure** **S1**. The simulated LCP wave to RCP wave converted efficiency (blue line) and phase delay (red line) of the micro-columns with different rotation angle at 0.75 THz (a), 0.8 THz (b), 0.85 THz (c), 0.9 THz (d). The inset in Figure S1(a) illustrates a micro-column with the rotation angle of 135 º.

**S2. Interference patterns for Q-PVBs generated by metasurface IV**

Metasurface IV is designed for four-channel Q-PVBs generation with topological charge of *l* = -1, 1, 2, -2. To identify the topological charges of the Q-PVBs, we calculated the interference pattern between the Q-PVB with topological charge *l* = 1 and the vortex beams generated by Metasurface IV. Figure S2(a) displays the calculated intensity distribution of proposed metasurface IV at the propagation distance of *z* = 15 mm, and interferometry patterns between the Q-PVB with topological charge *l* = 1 and the four generated Q-PVBs are shown in Figures S2(b)-S2(e). It can be observed that the continuous ring structure breaks into several spiral branches in the interferometry patterns and the number of spiral branches is equal to |*l_t_* – 1|, here *l_t_* represents the topological charge of Q-PVBs under test, thus we can identify different Q-PVBs by examining the number of spiral branches.


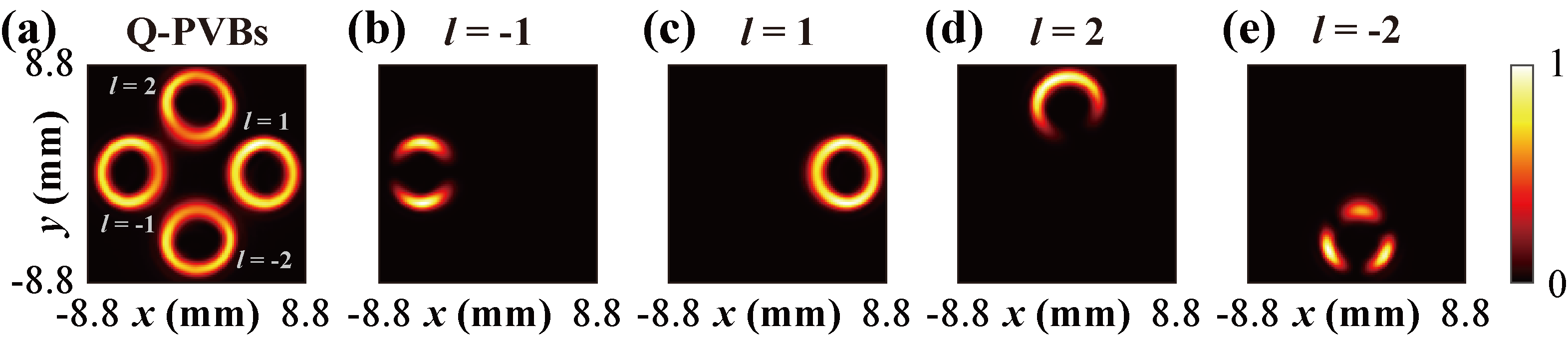


**Figure S2**. Recognition of the topological charges of the Q-PVBs. (a) The calculated intensity distribution of Q-PVBs at the propagation distance of *z* = 15 mm. (b)-(e) The calculated interferometry patterns between the Q-PVB with topological charge *l* = 1 and the Q-PVBs generated by metasurface IV.

**S3. Metasurfaces proposed to generate four-channel and six-channel focused vortex beams**

We proposed metasurface V and VI to generate four-channel (*l* = 1, -1, -1, 1) and six-channel vortex beams (*l* = 1, 1, 1, 1, 1, 1) at 0.75 THz. The focus length of these two metasurfaces were selected as *f* = 15 mm and *f* = 20 mm, respectively. For four vortex beams generation, the period of phase gradient is *P* = 3.45 mm; while for six vortex beams generation, the periods of phase gradient in *x*-direction and y-direction were set to *P* = 2.6 mm and *P* = 5.2 mm, respectively. Figures S3(a) and S3(d) display the partial enlarged optical images of the two metasurfaces. From Figures S3(b) and S3(e), the typical hollow ring-shaped intensity distribution and spiral phase distribution of four-channel and six-channel vortex beams are observed. Also, the good agreement between the calculated and measured results could be seen from Figures S3(c) and S3(f), which indicates the multifunctional metasurfaces have potential to be used to generate vortex beams arrays.


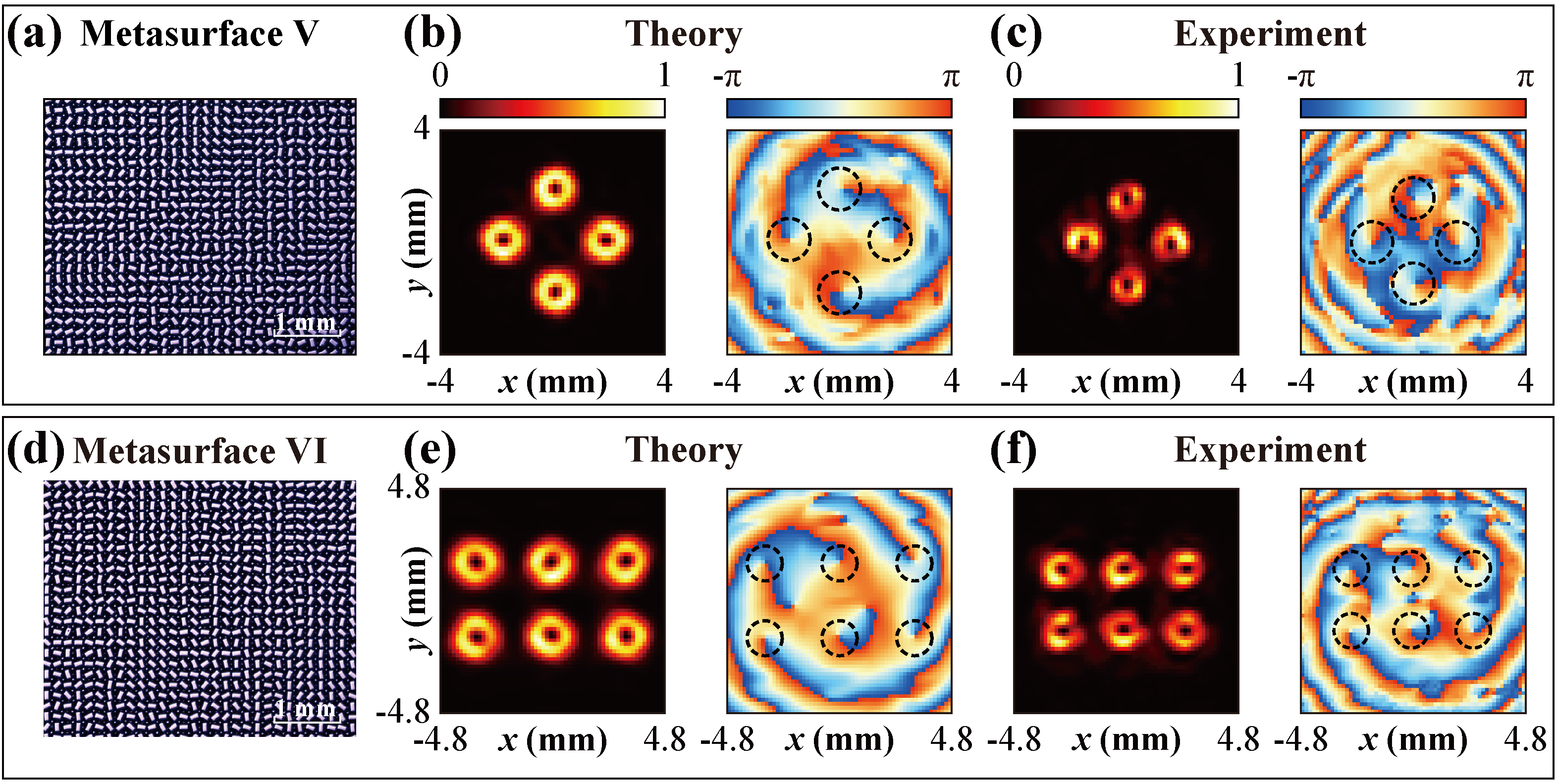


**Figure S3**. Four-channel and six-channel vortex beams generation. (a) and (d) Partially enlarged image of the fabricated metasurfaces. (b) and (e) Calculated intensity distributions and phase distributions of focused vortex beams at the focal plane (*f* = 15 mm for metasurface V and *f* = 20 mm for metasurface VI). (c) and (f) Measured intensity distributions and phase distributions of focused vortex beams at the actual focal plane (*f* = 12.5 mm for metasurface V and *f* = 16 mm for metasurface VI).

**S4. Metasurfaces proposed to generate four-channel and six-channel Q-PVBs**

We designed metasurface VII and metasurface VIII for four-channel and six-channel Q-PVBs generation at 0.75 THz. All the topological charges of generated Q-PVB are set to *l* = 1. Figures S4(a) and S4(b) show the schematic of the proposed metasurfaces. Considering the limited scanning area of our experimental setup, the period of the negative axicon is selected as *D* = 2.5 mm and *D* = 4 mm since the ring diameter decreases with the increment of *D*. Moreover, the focal length of metasurface VII and VIII are set as *f* = 15 mm and *f* = 20 mm, respectively. For four Q-PVBs generation, the period of phase gradient is *P* = 1.2 mm; while for six Q-PVBs generation, the period of phase gradient in *x*-direction and y-direction are set to *P* = 1.31 mm and *P* = 2.23 mm, respectively. As shown in Figures S4(c)-S4(f), four-channel and six-channel Q-PVBs with the annular rings can be seen from the good agreements between the calculated and experimental results. However, for six-channel Q-PVBs generation, due to we selected the phase gradient and focal length suitable for the limited scanning range of near-field setup, the doughnut shapes of Q-PVBs distort a little.


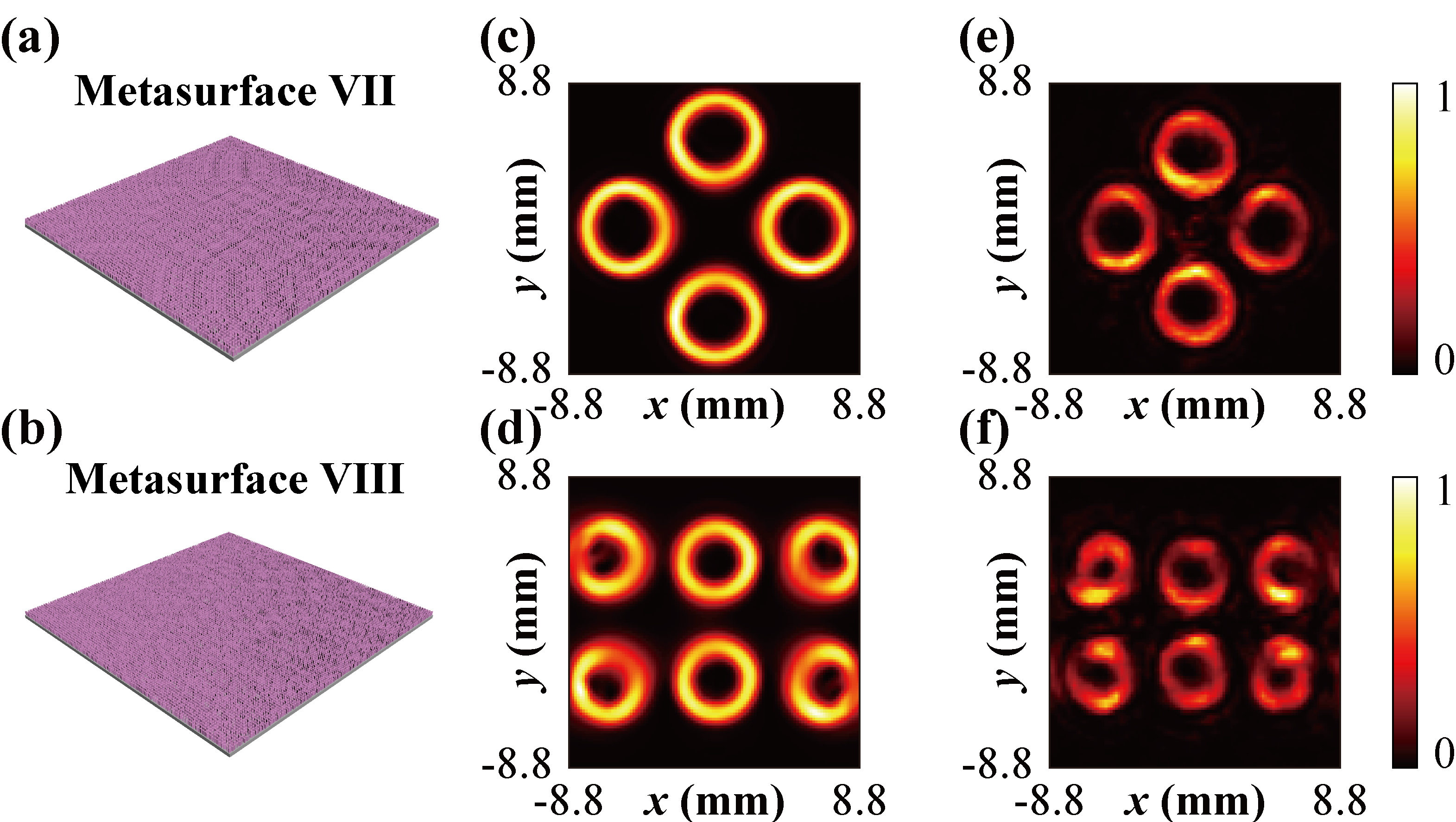


**Figure S4**. Four-channel and six-channel focused Q-PVBs generation. (a) and (b) Schematic of the proposed metasurfaces VII and VIII. (c) and (d) Calculated field intensity distributions of Q-PVBs at the focal plane (*f* = 15 mm for metasurface VII and *f* = 20 mm for metasurface VIII). (e) and (f) Measured field intensity distributions at the plane of *z* = 12.5 mm and 16 mm.

**S5. The relationship between the ring radius of Q-PVB and the two parameters**

To realize arbitrary manipulation of the ring radius of the generated Q-PVB, we explored the relationship between the ring radius of Q-PVB and the two parameters. Figure S5 is the calculated cross sections of annular intensity rings at *z* = 20 mm. The work frequency of the metasurfaces for the generation of Q-PVBs are set to 0.75 THz. Figure S5(a) show the relationship between ring radius *ρ* and the period of negative axicon *D*. Here the topological charge is *l* = 3, the focal length is *f* = 20 mm. From the calculated results, it can be seen that $\rho\propto1/D$. Figure S5(b) show the relationship between ring radius *ρ* and the focal length *f* (*l* = 3, *D* = 1.5 mm), from the calculated results we can observe that $\rho\propto f$.


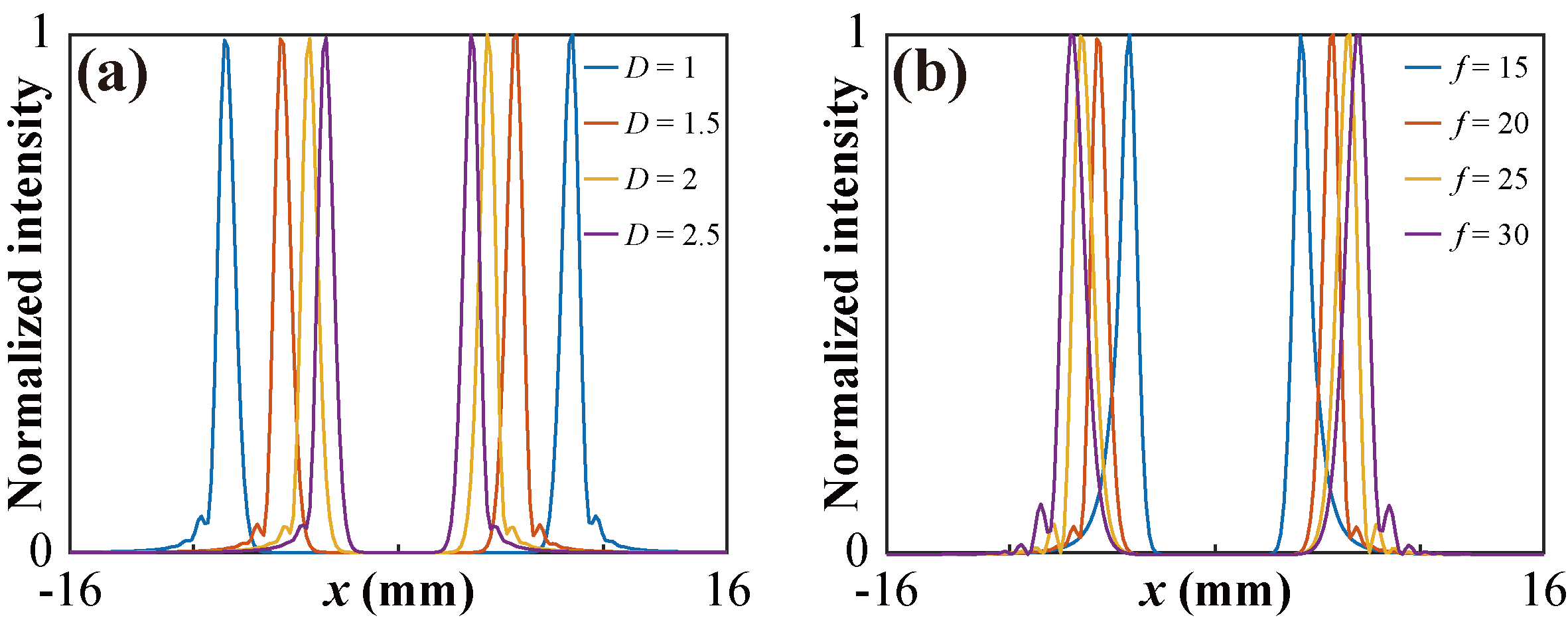


**Figure S5**. Calculated cross sections of Q-PVBs with topological charge of *l* = 3 at 0.75 THz. (a) The cross sections of intensity rings at propagation distance *z* = *f* = 20 mm with negative-axicon period *D* = 1, 1.5, 2, 2.5 mm. (b) The cross sections of intensity rings for *D* = 1.5 mm and *f* = 15, 20, 25, 30 mm at propagation distance *z* = 20 mm.

**S6. The conversion relationship between Q-PVB and BG beam**

We know that the perfect vortex beam can maintain the annular intensity distribution within a limited transmission distance and convert to the Bessel-Gaussian beam when the propagation distance much longer than the Rayleigh length. Figure S6 is the calculated intensity distribution of Q-PVB with topological charge of *l* = 3 at 0.75 THz. In Figures S6(a)-S6(c), the period of the negative axicon is selected as *D* = 1.5 mm, the focal length changes from *f* = 20 mm to *f* = 30 mm. From the calculated results, it can be observed that Q-PVB converts to Bessel-Gaussian beam after transmitting a distance of *r*_s_, and $r_{\text{s}}\propto f$. Furthermore, we fixed the focal length *f* = 20 mm and changed the negative axicon period in Figures S6(d)-S6(f). From the normalized field intensity profiles of *y* = 0 mm plane for *D* = 2, 1.5, 1 mm, it can be seen that $r_{\text{s}}\propto1/D$.


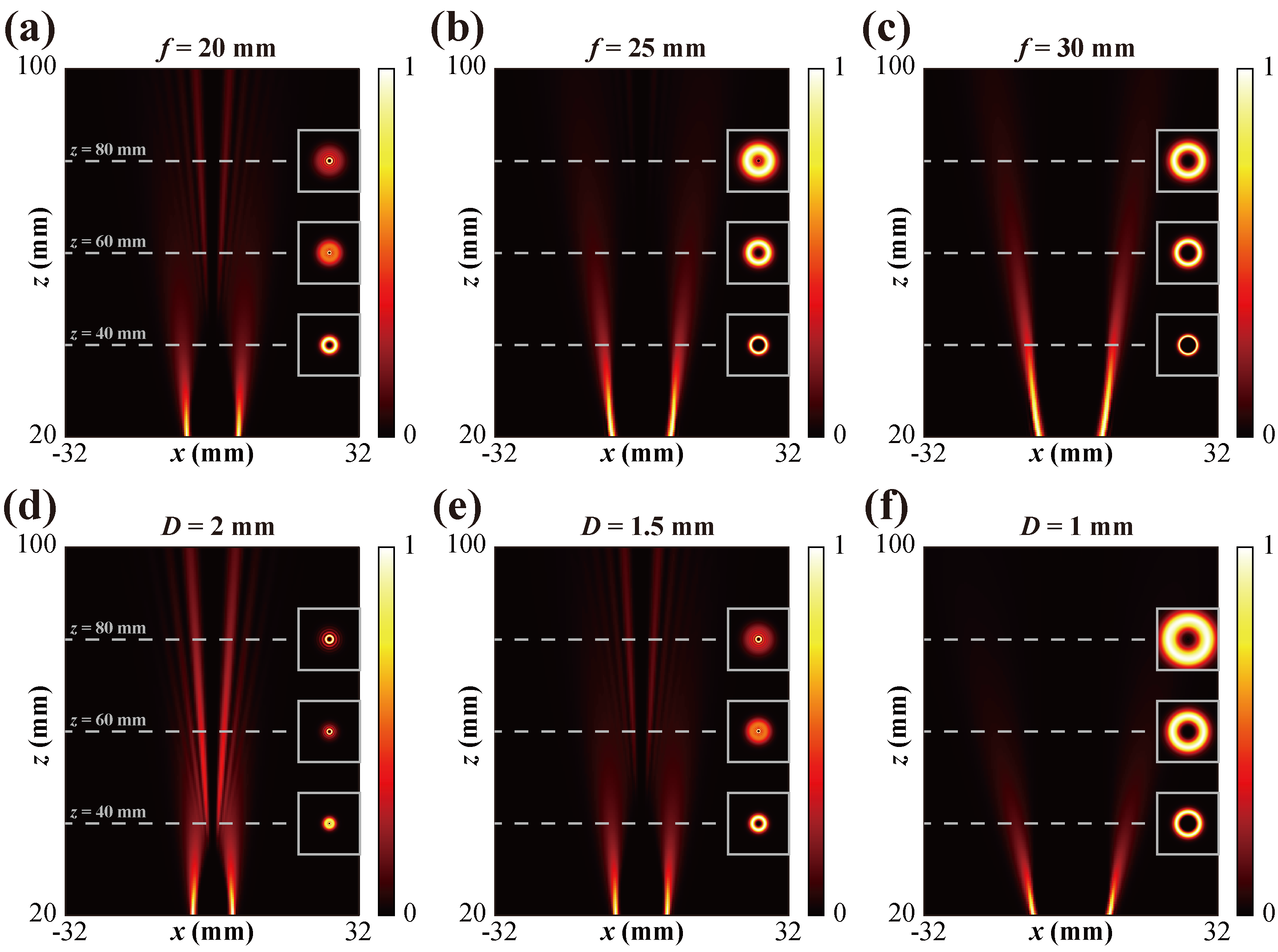


**Figure S6**. Calculated intensity distributions of Q-PVBs within a propagation distance of *z* = 80 mm. (a-c) Normalized field intensity profiles of *y* = 0 mm plane for *f* = 20, 25, 30 mm. (d-f) Normalized field intensity profiles of *y* = 0 mm plane for *D* = 2, 1.5, 1 mm. The topological charge of Q-PVB is *l* = 3 and the working frequency of all the metasurfaces is set to 0.75 THz. The insets in each figure correspond to the normalized intensity distribution of Q-PVB at the plane of *z* = 40, 60, 80 mm, respectively.
